# Acute Renal Failure after Abdominal Trauma: Renal Artery Spasm Hypothesis in Ischemic Infarction in a 12-Year-Old Girl

**DOI:** 10.1155/2020/6548591

**Published:** 2020-09-15

**Authors:** Aphaia Roussel, Jean-Daniel Delbet, Tim Ulinski

**Affiliations:** ^1^Pediatric Nephrology, DMU Origyne, APHP, Hôpital Armand-Trousseau, Paris, France; ^2^Sorbonne University, Paris, France

## Abstract

Posttraumatic renal failure is often due to postischemic renal infarction, caused by identified vascular lesions. In our patient, a 12-year-old girl with acute anuric renal failure requiring hemodialysis after severe abdominal trauma, no vascular lesion or thrombosis was identified. Nevertheless, CT-scan and renal biopsy showed typical lesions of diffuse bilateral renal ischemic necrosis. The main hypothesis is a severe bilateral arterial vasospasm after a blunt abdominal trauma. The patient recovered only partially with persisting chronic renal failure.

## 1. Introduction

Posttraumatic renal failure, although rare in paediatrics, is often due to severe trauma with postischemic renal infarct, caused by identified lesions such as aortic or renal artery dissection, bilateral renal vessel thrombosis, haemorrhagic shock, or Crush syndrome with rhabdomyolysis. We report on a 12-year-old girl with acute anuric renal failure requiring hemodialysis after abdominal trauma without identification of an underlying traumatic vascular lesion.

## 2. Case Presentation

Our patient, a 12-year-old girl (160 cm, 45 kg), presented at the emergency department for febrile abdominal pain with repeated vomiting after a violent physical aggression 12 hours before. An adolescent boy (175 cm, 60 kg) tied up the patient and jumped on her belly and kicked her several times. Heart rate and blood pressure were in the normal range.

The first blood tests showed increased plasma lipase (10 N) and liver cytolysis (10 N) without cholestasis or haemostasis disorders, suggesting acute posttraumatic pancreatitis. Furthermore, biological results showed metabolic acidosis with increased anion gap and normal plasma lactate level and acute renal failure (BUN: 25 mmol/L, serum creatinine: 503 umol/L). There were no electrolyte disturbances, but there were proteinuria (240 mg/mmol creatU) and hypoalbuminaemia (25 g/L), normal phosphate and calcium levels, increased LDH (3826 UI/L) and CPK (924 UI/L), and microscopic hematuria without myoglobinuria. There was no evidence for abdominal compartment syndrome.

She had normocytic anemia (Hb: 10.4 g/dL), without schistocytes, and elevated inflammatory markers (CRP: 311 mg/L and leucocytes: 24 × 10^9^/L including 20 × 10^9^/L neutrophils and fibrinogen at 7.1 g/L).

**Figure 1:**
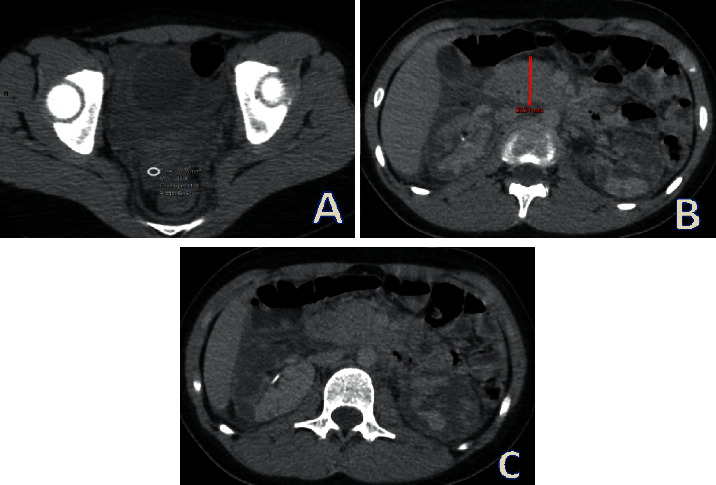
Abdomino-pelvic CT scan performed 12 hours after trauma, axial view. (a) Liquid of similar density as blood: haemoperitoneum. (b) Increase of pancreatic head size: pancreatitis. (c) Heterogeneous renal parenchyma with decreased enhancement during venous phase indicating ischemic lesions and necrosis.

A CT scan performed 12 hours after initial presentation (Figure 1) revealed a haemoperitoneum without pneumoperitoneum, an increase of pancreatic head size, and a heterogeneous and bilateral diminished enhancement of the kidney, suggestive of bilateral renal necrosis.

**Figure 2:**
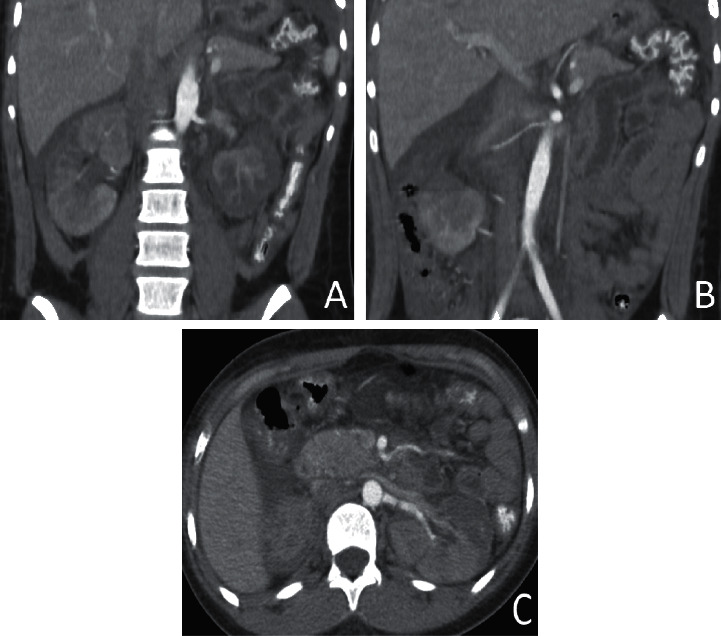
CT scan performed on day 7. (a) Coronal view, arterial phase; renal arteries are both clearly identified, without dissection or thrombosis. (b) Coronal view, arterial phase, 3 right renal arteries, anatomical variant. (c) Axial view, probable unilateral artefact without any flow interruption.

A second CT scan was performed on day 7 (Figure 2), showing a bilateral pleural, pericardial, and peritoneal effusion and persistence of lesions compatible with infarction in multiple zones of the renal parenchyma, with a patchy distribution. No artery dissection and no thrombosis were identified. A unilateral sign of left renal arterial thrombosis was suspected but identified as an artefact because there was no blood flow interruption.

A Doppler sonography on day 15 confirmed the integrity of the blood flow, especially at the renal artery ostium. Doppler sonography was repeated several times during the first three months after initial presentation and blood flow remained normal.

As no causative lesion could be identified on vascular CT scans, we performed a renal biopsy on day 7, which revealed complete ischemic necrosis of the renal parenchyma, affecting the proximal and distal tubules, but also the glomeruli and interstitial oedema.

A complete immunologic and viral work-up was negative. Renal failure with oliguria and hyperkalemia (creatinine max: 700 umol/L on day 5) required three hemodialysis sessions between day 5 and day 13. On day 18, she became polyuric requiring IV hydration. We noted a progressive improvement of her renal function with a stable serum creatinine level at 120 umol/L (eGFR: 65 ml/min/1.73 m^2^) after three months with normal blood pressure.

Abdominal pain decreased until day 30, and plasma lipase normalized after three months.

A thrombophilia work-up was performed after normalisation of inflammation parameters, and it showed an isolated deficiency in S protein (25%).

Ultrasound of the supra-aortic trunks and cardiac ultrasound were performed in order to rule out an embolic cause for renal ischemic necrosis. Preventive anticoagulation was started after confirmation of the S protein deficiency.

## 3. Discussion

Renal infarcts are rare with an estimated incidence of 0.007% in a prospective study with a mean age of 64 years [1] and 0.004% in another with a mean age of 60 years [2]. Around 30% of patients progress towards chronic renal failure [3].

Paediatric cases remain anecdotal, and there are no studies of the frequency of renal infarction in children. In cases of trauma, the renal infarct can be unilateralor bilateral, not always being responsible of renal failure. The CT scan allows the diagnosis with typical images of hypodensity and the absence of renal cortical enhancement. Identification of a vascular lesion responsible for ischemia and infarct is frequent. For the patients exposed to abdominal trauma, it can be classified avulsion, laceration, dissection, and occlusion of the renal artery [4]. Among these, the most frequent are renal artery dissections [5]. Others describe lesions due to Crush syndrome or haemorrhagic shock.

Traumatic dissection of the renal artery is often due to a mechanism of acceleration or/and deceleration with a closed abdominal trauma [6] and is easily identified on CT scan. Thrombosis of the renal artery is more often unilateral than bilateral. Its incidence in renal trauma is estimated around 2% [7]. S protein deficiency is rarely identified in renal artery or venous thrombosis. In our case, we could not identify any thromboembolic lesion.

Absence of myoglobinuria ruled out rhabdomyolysis. No dissection or arterovenous shunt was identified on CT scan. However, CT scan revealed typical images for bilateral renal infarcts. One hypothesis would be the occlusion of renal arteries by compression against the spine bone. This mechanism has already been reported but is often associated with a posttraumatic thrombosis [8]. We hypothesize that this trauma may provoke vasospasms within the renal parenchyma or in the renal artery, responsible for a deep ischemia. A vasospasm of small vessels and liberation of toxin with consequent endothelial injury has been described as the initiating event in the process of cortical necrosis [9].

Abdominal vasospasm can be encountered during endovascular procedures or after blunt abdominal trauma. This mechanism is also well known in abdominal surgery and particularly in neuroblastoma surgery [10].

This hypothesis was presumed in a case report [11] and clearly established in another one after a severe trauma in a 4 year-old child [12]. A CT scan one hour after the accident showed a vasospasm of the right renal artery and a typical image of renal ischemia. However, CT scan was controlled five hours later and was normal without any vascular abnormality.

Our patient had a CT scan performed 12 hours after the trauma, presumably too late to visualize the vasospasm. Therefore, we hypothesize that bilateral transitory blood flow interruption via vasospasm was responsible for the renal necrosis.

If renal artery spasm is considered responsible, it may avoid unnecessary or invasive investigations. Retrospectively, we have to admit that a kidney biopsy was not necessary for our patient management. We performed a kidney biopsy, because of the absence of identified vascular lesions. If a CT scan had been performed earlier in our patient and an arterial vasospasm with blood flow interruption was identified, the distinction from thrombotic occlusion or intimal flap formation would have been of importance because in this situation, the management would have been very different and would have required vascular intervention.

Functional mechanisms, such as vasospasms, should be suspected, if no lesion can be identified [13]. This mechanism is also well known in abdominal surgery and particularly in neuroblastoma surgery [10].

The cause of renal infarct in our patient remains uncertain, but bilateral posttraumatic vasospasm is the most plausible hypothesis.

## 4. Conclusion

Posttraumatic bilateral renal infarcts are rare and often due to identified lesions, especially renal artery dissection and renal artery thrombosis. Even though the precise cause of the renal infarct in our patient remains uncertain, a bilateral renal vasospasm should be considered, because there were no identified vascular lesions and no systemic hypoperfusion due to hypotension or shock.
